# Imaging Characteristics of Occipital Bone Osteoblastoma

**DOI:** 10.1155/2013/930623

**Published:** 2013-07-17

**Authors:** Adam Alli, Philip Johnson, Alan Reeves

**Affiliations:** Department of Neuroradiology, University of Kansas Medical Center, 3901 Rainbow Blvd., Kansas City, KS 66160, USA

## Abstract

Osteoblastoma is a rare benign tumor of the calvarium. We present the case of a 20-year-old female with occipital osteoblastoma and discussion of imaging modalities of calvarial osteoblastoma. To our knowledge, this is the ninth reported case of occipital osteoblastoma. Imaging characterization of osteoblastoma may vary. Plain radiograph, CT, MRI, and CT angiography establish osteoblastoma characterization and vascular supply prior to surgical resection.

## 1. Introduction

Benign osteoblastoma was initially discovered separately by Jaffe and Lichtenstein in 1956 [1] and is a primary bone lesion that is usually associated with the vertebral column and the long bones [2]. Calvarial osteoblastoma is rare, with a more common appearance in the frontal and temporal bones when it does occur [3]. Calvarial osteoblastoma is essentially similar to osteoblastoma in traditional locations [4].

Multiple imaging modalities are traditionally used to evaluate calvarial lesions. Imaging characteristics of osteoblastoma may vary and result in difficult establishment of the radiologic diagnosis in the rare location of the calvarium. Osteoblastoma is treated with surgical resection as well as prior embolization to minimize intraoperative hemorrhage [5]. We report the case of an occipital bone osteoblastoma with discussion of imaging characteristics and radiologic modalities to best characterize osteoblastoma of the calvarium.

## 2. Case Report/Technique

A 20-year-old female presented with right occipital tenderness, headache, and cervical pain. The patient's past medical history was essentially unremarkable apart from a remote history of mononucleosis. Conservative medical therapy, including muscle relaxants and anti-inflammatories, was unsuccessful in relieving the patient's symptoms. Physical exam demonstrated tenderness in the right suboccipital region. Neurological exam was within normal limits. The patient subsequently underwent imaging workup of her head.

Plain radiograph demonstrated a mixed sclerotic and lytic lesion extending from the inner table of the occipital bone protruding into the right posterior fossa. CT angiogram of the head depicted a patchy heterogeneous enhancing 3.2 × 4.1 cm expansile calvarial mass in the right occipital region with cortical erosion of the outer cortex, multiple tiny cystic areas, and some bony matrix. There was no obvious intracranial blood supply to the mass. MRI showed low signal intensity on the T1-weighted images, mixed high and low signal intensities on the T2 and FLAIR images, and postgadolinium enhancement. 

Prior to resection of the neoplasm, the lesion was embolized using a combination of intra-arterial particles and coils. The arteriogram revealed a hypervascular occipital bone tumor supplied by a dilated occipital branch from the right external carotid artery and a dilated muscular branch from the distal right vertebral artery. Following embolization, the tumor was surgically resected with minimal blood loss. Histopathology diagnosis revealed osteoblastoma.

## 3. Discussion

Osteoblastoma was initially described in 1956 [2, 3, 5] and is defined as a vascular, osteoblastic, and nonfibroblastic tumor [2, 6]. Osteoblastoma is considered a predominately intramedullary process [7] and accounts for approximately 1% of primary bone tumors [3, 6]. Osteoblastomas most commonly occur in the vertebral column and long bones [2] with rare occurrence in the calvarium [2, 3, 5, 6]. They typically demonstrate mixed lytic and sclerotic components with bony destruction and a well-circumscribed sclerotic border [2, 3, 5, 6]. Surgical resection remains the definitive treatment for benign osteoblastoma due to recurrence and risk of malignant transformation [1, 3]. Imaging characterization of these lesions is an integral part in management of these patients.

**Figure 1:**
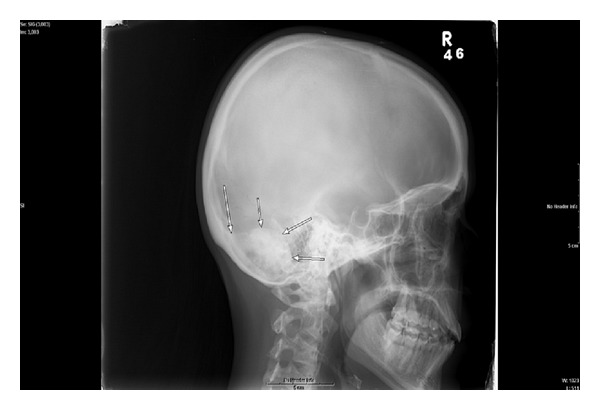


Plain radiography should demonstrate a well-circumscribed expansile mass with a mixed lytic and sclerotic appearance [2, 3, 5]. There may be a predominant lytic or sclerotic component and it may be difficult to differentiate from osteoid osteoma. There have been reported cases of osteoblastoma with aneurysmal bone cyst component histopathologically [8, 9]. Due to the intermedullary nature of osteoblastoma, plain radiograph should demonstrate at least the expansile component of the lesion. Rarely, periosteal osteoblastoma can occur which can mimic a meningioma [7]. In contrast, osteosarcomas will likely have a much more aggressive and destructive appearance [3]. On plain radiograph our case shows a mixed sclerotic and lytic lesion extending from the inner table of the occipital bone protruding into the right posterior fossa (Figure 1).

**Figure 2:**
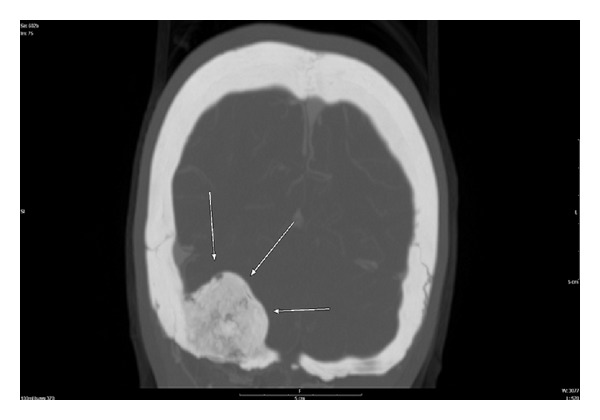


While plain radiographs can provide useful information, most cases will be diagnosed using CT. On CT, osteoblastoma can demonstrate a mixed sclerotic and lytic intraosseous lesion with a well-circumscribed sclerotic border as well as bony destruction [2, 3, 5]. Contrast enhancement is variable on CT [6]. Our case shows a patchy heterogeneous enhancing 3.2 × 4.1 cm expansile calvarial mass in the right occipital region with cortical erosion of the outer cortex, multiple tiny cystic areas, and some bony matrix (Figure 2).

Geographic definition and osseous characteristics are well established by CT.

**Figure 3:**
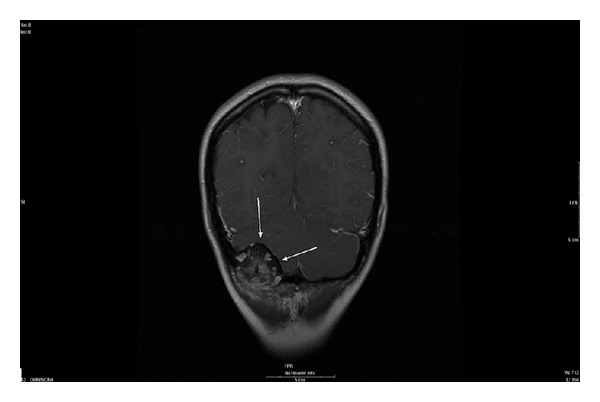


Surgical planning for resection of the tumor is dependent on vascular supply and involvement of the adjacent soft tissues. MRI provides important information on surrounding brain parenchyma as well as the lesion itself. Osteoblastoma signal characteristics are highly variable, ranging from hypointesity on T1-weighted images with hyperintensity on T2-weighted images to hypointensity on both T1- and T2-weighted images [2, 3, 5, 6]. Gadolinium enhancement is varied as well with reports of enhancing osteoblastomas to nonenhancing osteoblastomas [3]. While characterizing the lesion is variable on MRI, the extension of the tumor and involvement of the adjacent soft tissues should be the role of MRI [1, 3, 5]. However, when the bone marrow is involved, osteoblastoma and edema may have similar MRI characteristics [1]. Our case depicts low signal intensity on the T1-weighted images, mixed high and low signal intensities on the T2 and FLAIR images, and postgadolinium enhancement (Figure 3).

Magnetic resonance imaging is the modality of choice for determination and description of osteoblastoma extension as well as involvement of the adjacent soft tissues [1, 3, 5, 10].

**Figure 4:**
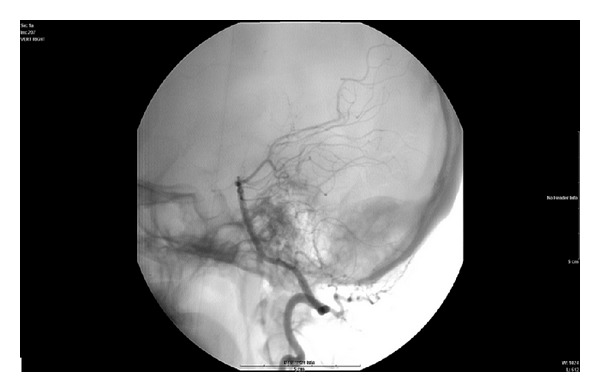


In addition to the involvement of the adjacent soft tissues, vascular supply is an important factor in treatment of osteoblastoma. CT angiography helps determine probable vascular supply to the tumor and can help preoperative embolization. Our case showed no obvious intracranial blood supply to the mass on CT angiography. Conventional angiography at embolization demonstrated hypervascular occipital bone tumor supplied by a dilated occipital branch from the right external carotid artery and a dilated muscular branch from the distal right vertebral artery (Figure 4).

Additional imaging which can be considered is ^18^F-FDG-PET and technetium-99 scintigram. Interestingly, despite its benign nature, osteoblastoma has been reported to have increased uptake for these modalities [6].

## 4. Conclusion

Osteoblastoma is a rare benign tumor of the calvarium. With surgical resection as the definitive treatment, imaging characterization plays a vital role in diagnostic workup and treatment planning. Plain radiograph can provide useful information, but most cases will be diagnosed with CT. CT and MRI in combination are useful for describing the osseous lesion and involvement of adjacent soft tissues. CT angiography may be a useful tool prior to embolization and eventual resection of the tumor.

## Abbreviations

CT: Computed tomography
MRI: Magnetic resonance imaging
FLAIR: Fluid attenuation inversion recovery
^18^F-FDG-PET: Fluorine 18 fluorodeoxyglucose positron emission tomography.
